# The gig satisfaction gap: a comparative machine learning analysis of job satisfaction determinants between gig and traditional employees

**DOI:** 10.3389/fpsyg.2026.1871662

**Published:** 2026-09-03

**Authors:** Yeye Li, Jinkai Cheng

**Affiliations:** 1 School of Business Administration, Anhui University of Finance and Economics, Bengbu, China; 2 Business School, Sichuan University, Chengdu, China

**Keywords:** compensation, gig workers, job satisfaction, machine learning, work-life balance

## Abstract

**Introduction:**

Prior research has yielded inconsistent findings regarding whether gig workers experience lower job satisfaction than traditional employees, while the specific factors underlying these differences remain insufficiently understood. This study systematically compares job satisfaction between location-based gig workers and traditional employees and identifies the factors that characterize their respective work experiences.

**Methods:**

Using 19,610 online employee reviews collected between 2011 and 2025, we developed a machine-learning text classifier with 92.22% accuracy to distinguish reviews written by gig workers from those written by traditional employees. We then combined independent-samples *t*-tests, sentiment analysis, and topic modeling to compare satisfaction levels, emotional patterns, and the factors associated with job satisfaction across the two groups.

**Results:**

The results show that gig workers and traditional employees report comparable levels of work–life balance satisfaction, but gig workers exhibit significantly lower overall job satisfaction, with the largest disparity observed in compensation satisfaction. Textual analyses further reveal distinct patterns in the two groups’ work experiences. Gig workers express predominantly negative sentiment toward driver-related costs, job allocation, and wages, whereas traditional employees place greater emphasis on organizational culture, career growth, and employee benefits. Topic modeling identifies 25 factors associated with job satisfaction and highlights several gig-specific concerns, particularly driving costs and the allocation of work opportunities.

**Discussion:**

These findings help reconcile inconsistent evidence on the gig–traditional worker satisfaction gap by showing that the disparity is dimension-specific rather than uniform across all aspects of work. By identifying work arrangements and economic conditions that are particularly salient to location-based gig workers, this study extends understanding of job satisfaction in platform-mediated work and provides actionable implications for platform managers and policymakers seeking to improve gig workers’ work experiences.

## 1 Introduction

Advances in artificial intelligence (AI) technology have accelerated the digital transformation of the workplace, fundamentally altering how individuals work and how organizations manage their employees. In particular, there is a growing trend toward employment relationships that deviate from traditional permanent arrangements in favor of flexible employment models. This emerging form of employment no longer necessitates a fixed workplace or standard working hours. Consequently, organizations are increasingly relying on gig workers to enhance flexibility in job assignments and reduce expenses associated with traditional employment relationships. The impact of these evolving employment relationships on employees’ psychological outcomes has garnered significant scholarly attention. A fundamental question remains whether differences exist in work-related attitudes and behaviors between traditional workers and gig workers, given the latter group’s exposure to financial uncertainty, job insecurity, unpredictable career paths, job transience, and a lack of coworker interaction (Ashford et al., 2018).

Employee job satisfaction is defined as a positive emotional state resulting from the evaluation of one’s job or job experience (Thoresen et al., 2003). It is a critical factor influencing employee performance, turnover intention, and organizational commitment (Gutierrez et al., 2012; Hewagama et al., 2019; Sainju et al., 2021). Maintaining high levels of employee satisfaction is paramount for organizational success and employee well-being. Therefore, understanding the drivers of job satisfaction can yield insights that help companies make effective organizational decisions and gain a competitive advantage. Although extensive research on job satisfaction has been conducted over the years, previous studies have focused primarily on traditional employees, largely disregarding a specific group of professionals known as gig workers (Ashford et al., 2018; Barley et al., 2017).

Previous research on job satisfaction among gig workers and traditional workers has yielded conflicting results. Some studies indicate that gig workers report higher levels of job satisfaction than traditional workers, attributed to the flexibility and autonomy inherent in gig work (Berger et al., 2019). Gig workers can adjust their work time, workload, and methods to fit individual needs, allowing for a better balance between work and personal responsibilities, which leads to higher satisfaction (Berger et al., 2019; Friedman, 2014). Conversely, other studies suggest that gig workers experience lower job satisfaction because they lack genuine independence or autonomy (Ravenelle, 2019). Specifically, those in ride-sharing and delivery services are often not independent contractors as claimed by gig platforms. Instead, they are subject to work-related risks, such as vehicle wear and tear and fuel consumption, which are transferred onto them by the platforms (Goods et al., 2019). Furthermore, labor relations remain unclear, leaving gig workers unprotected and denied access to basic benefits such as minimum wage, health insurance, and unemployment insurance (Agrawal et al., 2022). Moreover, gig workers are subject to algorithmic monitoring and control, further exacerbating their dissatisfaction (Won et al., 2023). Consequently, the absence of genuine independence and autonomy leads to diminished job satisfaction and heightened feelings of insecurity.

Han and Wang (2022) argued that these conflicting views arise primarily from different research perspectives and the uncertain selection of research groups. While they used questionnaires to clarify some of these mixed findings, their focus was on self-employed workers rather than gig workers specifically. Therefore, further research is required to investigate the job satisfaction of gig workers distinctly.

The validity of using questionnaires to gain insights is frequently debated in research (Hardy and Ford, 2014). Questionnaires are limited because predefined questions can restrict insights beyond predetermined dimensions, and the validity and reliability of collected data may be compromised by inaccuracies or incomplete responses (Sainju et al., 2021). To address the limitations of data collection and research paradigms in traditional organizational research, new approaches are necessary. Big data methods can bridge these limitations (Tonidandel et al., 2018), and researchers are recommended to employ techniques such as data mining, data visualization, and machine learning to address various issues in organizational behavior research. By utilizing these methods, researchers can gather and analyze vast amounts of data to gain insights and make accurate predictions, leading to a deeper understanding of organizational behavior and improved decision-making.

Our study employs an innovative data source by analyzing online reviews written by employees, as opposed to relying solely on traditional employee surveys. Through the application of machine learning techniques on online reviews collected from Glassdoor^1^, we developed a classifier to distinguish between gig workers and traditional workers based on the textual content of the reviews. This approach allowed us to conduct a comprehensive investigation into the differences in job satisfaction between gig workers and traditional workers. Building upon this investigation, we employed statistical analysis, sentiment analysis, topic modeling, and visualization techniques to further explore and comprehend variations between the two groups. To evaluate and interpret the potential topics identified, our study also utilizes topic visualization techniques. Overall, our study offers a unique perspective on understanding the satisfaction of gig workers and highlights important factors that organizations can focus on to improve job satisfaction and reduce turnover.

## 2 Literature review

### 2.1 The gig economy and psychological disparities

The proliferation of the gig economy represents a paradigm shift in the contemporary labor market, fundamentally altering the psychological contract between workers and organizations. Compared with traditional employees, gig workers ostensibly enjoy greater autonomy and flexibility, allowing them to dictate their schedules and work environments. However, this perceived freedom often masks a darker reality characterized by income volatility, pervasive job insecurity, unclear career progression paths, and profound social isolation (Ashford et al., 2018). This paradox of autonomy suggests that while gig workers are free from direct supervision, they are simultaneously exposed to heightened economic precarity. Platform companies such as Uber and Lyft increasingly rely on algorithmic management to oversee hiring, performance evaluation, pay structures, and termination processes (Kellogg et al., 2020). This digital surveillance creates a “black box” of management where decisions are opaque, potentially heightening workers’ anxiety and frustration due to a lack of procedural justice and human interaction (Rahman, 2021).

Existing literature has begun to examine gig workers’ well-being, psychological contracts, and behavioral responses to leadership and incentives (Berger et al., 2019; Cropanzano et al., 2023; Fest et al., 2021). These studies highlight the unique stressors associated with platform work, including the constant pressure to maintain high ratings and the absence of traditional safety nets. However, a significant methodological limitation persists: current research relies largely on traditional survey or experimental data. Such methods are often constrained by self-report biases, limited sample sizes, and the inability to capture spontaneous, unprompted employee sentiments. This underscores the critical need for alternative data sources, such as employee online reviews, which offer a more ecologically valid window into the lived experiences of gig workers without the interference of researcher-induced prompts.

### 2.2 Employee online reviews as unstructured big data

Advances in AI and digital communication technologies have fueled an explosion of user-generated content (UGC), with online reviews emerging as a key information source for organizational research (Jung and Suh, 2019). Unlike structured surveys, employee online reviews represent organic expressions of sentiment, capturing immediate reactions to workplace events and conditions. Management scholars are increasingly leveraging these data to assess organizational culture, predict firm outcomes, and evaluate diversity and inclusion practices (Schmiedel et al., 2019; Symitsi and Stamolampros, 2021; Wang et al., 2023). Employee reviews describe what workers genuinely like and dislike about their employers, making them well suited to identifying the authentic drivers of job satisfaction that might be overlooked in predefined questionnaire items.

However, the unstructured nature of such text poses significant analytic challenges, particularly for researchers lacking advanced technical skills or computational resources (Symitsi et al., 2021; Guo et al., 2024). Textual data is often noisy, containing sarcasm, idioms, and context-dependent meanings that traditional content analysis cannot easily decipher. To overcome these barriers, recent work demonstrates the usefulness of Natural Language Processing (NLP) techniques, including topic modeling, word embeddings, and text classification, for extracting nuanced insights from these large-scale data collections (Hannigan et al., 2019; Schmiedel et al., 2019; Speer, 2021; Fyffe et al., 2024; Kobayashi et al., 2018a). Building on this stream of research, our study employs a comprehensive suite of machine learning techniques—including classification modeling, sentiment analysis, and structural topic modeling—to systematically extract and analyze the factors that influence job satisfaction from the online reviews of gig workers, thereby bridging the gap between big data potential and organizational theory.

### 2.3 Machine learning methodologies in organizational research

#### 2.3.1 Text classification for worker identification

Text classification is a supervised machine learning technique that involves training a model on a labeled dataset to automatically assign predefined categories or labels to unlabeled text. This technique has gained significant importance in the field of organizational behavior research, enabling researchers to analyze and comprehend large volumes of textual data efficiently and accurately. For example, Kobayashi et al. (2018a) provided a seminal example of text classification, wherein they developed a model using machine learning techniques to predict the category of required skills in job postings, achieving an accuracy of 89%. Similarly, Fyffe et al. (2024) utilized machine learning techniques to classify the Big Five personality traits from text, yielding an accuracy of 83%. In the context of this study, text classification is critical for distinguishing between gig workers and traditional employees within a mixed dataset of online reviews. By accurately labeling the worker type based on textual cues, we ensure that subsequent analyses compare distinct groups, thereby validating the comparative framework of our research design.

#### 2.3.2 Sentiment analysis for emotional valence

Sentiment analysis evaluates the emotional valence expressed in text and is widely applied in marketing, social media, and consumer research to gauge public opinion (Jain et al., 2021; Nilashi et al., 2018). In the employment context, it helps organizations understand employees’ attitudes and satisfaction levels beyond simple numeric ratings. Bajpai et al. (2023), for instance, conducted aspect-level sentiment analysis of employee reviews using sentiment analysis, revealing distinct patterns in evaluations of pay and work culture across different industries. Zhang et al. (2021) used Twitter data to examine employees’ attitudes toward remote work during the COVID-19 pandemic, highlighting how external crises shift sentiment dynamics. By quantifying sentiment, researchers can capture employees’ emotional states and perceptions of their work experiences at scale. Our study leverages this technique to measure the intensity of positive or negative emotions associated with specific job facets, allowing for a granular comparison of how gig workers and traditional employees emotionally respond to similar organizational stimuli.

#### 2.3.3 Topic modeling for thematic exploration

Topic modeling is an unsupervised machine learning technique used to uncover the underlying topics or trends in large unstructured data collections without prior labeling. The Latent Dirichlet Allocation (LDA) model is the most commonly used approach for topic modeling. Hannigan et al. (2019) and Kobayashi et al. (2018b) outlined LDA applications and demonstrated how topic modeling can be used to analyze and visualize job postings, while Won et al. (2023) used topic models to examine gig workers’ experiences with algorithmic management. However, standard LDA assumes topic independence and does not directly model the effect of covariates on topics, limiting its utility for hypothesis testing (Roberts et al., 2019). Structural Topic Modeling (STM) addresses these limitations by allowing topic correlations and estimating how covariates (such as worker type) influence topic prevalence and word usage. Management scholars have used STM to mine leadership challenges (Tonidandel et al., 2022), explore platform workers’ responses to new forms of organizing (Karanović et al., 2021), and examine gender bias in developmental feedback (Doldor et al., 2019). In our study, we apply STM to compare dominant factors in reviews written by gig workers versus traditional workers. This advanced approach not only identifies what topics are discussed but also statistically tests how the prevalence of these topics differs between groups, thereby identifying how the two groups differ in what they value at work and providing a robust empirical basis for our theoretical contributions.

## 3 Data and methodology

### 3.1 Research design and objectives

To comprehensively investigate the nuances of job satisfaction within the evolving landscape of work, this study adopts a robust data-driven approach integrating text classification, sentiment analysis, and Structural Topic Modeling (STM). Leveraging a large-scale corpus of online employee comments from global platforms, our research design is structured to address three critical dimensions: (a) the current state of job satisfaction among gig workers compared to traditional employees; (b) the specific determinants driving satisfaction or dissatisfaction within each group; and (c) the divergent perceptions of these factors across employment types. Accordingly, our methodology is engineered to achieve four primary objectives: first, to accurately differentiate employee categories through supervised text classification; second, to conduct descriptive analyses that reveal underlying factual patterns; third, to quantify and compare the emotional valence expressed by gig versus traditional workers; and fourth, to utilize STM algorithms to isolate key factors influencing job satisfaction and examine structural differences between the two cohorts. The research framework comprises five sequential phases: data collection, preprocessing, representation, modeling, and evaluation, each detailed in the subsequent sections.

### 3.2 Data collection

The empirical data for this study were sourced from Glassdoor, a premier global platform for employee reviews and corporate insights. Renowned for its extensive reach, Glassdoor attracts over 55 million unique monthly visitors and hosts a vast repository containing more than 115 million reviews, salary reports, and interview insights spanning over 600,000 companies worldwide. This platform provides a rich, ecologically valid source of unsolicited employee feedback, minimizing the social desirability bias often present in traditional survey data. Specifically, this study focuses on employee reviews related to Uber, a paradigmatic example of a gig economy platform offering ride-hailing and delivery services. Following the data collection protocol outlined by Guo et al. (2024), we initiated the scraping process in January 2026, ensuring a contemporary and relevant dataset that captures recent shifts in labor dynamics.

Although online crowdsourced data can suffer from extreme-rating and selection biases, Glassdoor’s “Give-to-Get” mechanism empirically mitigates individual-level extreme scores (Marinescu et al., 2021). Furthermore, regarding representativeness concerns, a recent study demonstrates that such big online convenience samples yield highly reliable and robust metrics, serving as a valid and valuable empirical resource to supplement traditional probability surveys (Fleming et al., 2024).

### 3.3 Data preprocessing

Data preprocessing serves as the foundational step that significantly influences the reliability and validity of subsequent machine learning models. This stage is critical for reducing feature redundancy and optimizing corpus size, thereby enhancing the computational efficiency and analytical power of the study (Hickman et al., 2022). Our preprocessing pipeline involved several rigorous steps: conversion of text to lowercase to ensure uniformity, removal of punctuation and special characters, elimination of standard stop words to reduce noise, and lemmatization to reduce words to their base forms. Additionally, we filtered out extremely short reviews and non-English content to maintain data quality. By systematically cleaning the raw text, we ensured that the resulting dataset accurately reflects substantive employee sentiments rather than artifacts of writing style or formatting inconsistencies.

### 3.4 Data representation

Following preprocessing, transforming the textual data into a numerical format comprehensible to machine learning algorithms is essential. While the Bag-of-Words (BoW) model is a common approach that represents text by counting word frequencies, it fails to capture the relative importance and uniqueness of terms within the corpus. To overcome this limitation, we employed the Term Frequency-Inverse Document Frequency (TF-IDF) weighting scheme. TF-IDF effectively identifies words that are significant and unique to specific documents while downplaying terms that appear frequently across the entire dataset. This representation method improves the accuracy and interpretability of the resulting models by highlighting discriminative features that are most relevant to the topics of job satisfaction and employment conditions.

### 3.5 Modeling strategy

The modeling phase integrates classification and topic modeling techniques to extract meaningful insights from the represented data. A significant challenge in our dataset was that 19.3% of the collected reviews lacked explicit job title information, rendering it impossible to directly distinguish between gig workers and traditional employees based on metadata alone. To address this, we developed a binary text classification model trained on labeled subsets to predict employee categories based on linguistic patterns within the reviews. Once the employee categories were inferred, we proceeded to topic modeling to uncover latent themes. Unlike traditional Latent Dirichlet Allocation (LDA), which assumes topic independence, we utilized Structural Topic Modeling (STM). STM allows for the incorporation of document-level covariates (such as employee type), enabling us to statistically test how topic prevalence and content vary between gig workers and traditional employees. This approach provides a more nuanced understanding of how employment relationships shape the discourse around job satisfaction.

### 3.6 Model evaluation

Rigorous model evaluation is indispensable in machine learning and natural language processing (NLP) tasks to ensure the robustness and generalizability of findings. In this study, we employed a multi-metric evaluation framework tailored to each model type. For the text classification model, we assessed performance using precision, recall, F1 score, and accuracy to ensure balanced performance across classes, mitigating the risk of bias toward the majority class. For the topic models, we evaluated semantic coherence and exclusivity to ensure that the identified topics were interpretable and distinct. These evaluation metrics provide a comprehensive assessment of model performance, allowing for informed decisions regarding model selection and validation. Detailed methodological standards for these evaluations align with established practices in computational social science (Kobayashi et al., 2018a; Roberts et al., 2019).

## 4 Results

### 4.1 Overview of data

The data employed in this study were sourced from English-language reviews on Glassdoor. The dataset comprises 19,610 reviews, encompassing reviewer metadata (e.g., job title, employment status, and review timestamp) and textual content, including overall summaries, company pros, and cons. Additionally, numerical ratings ranging from 1 to 5 were collected across an overall dimension and six sub-dimensions; detailed specifications are provided in Table 1.

**Table 1:** Summary of missing values and explanations in employee reviews dataset.

Column name	Missing values	Explanation	Data type
Review-ID	0	Unique identifier for each review	Numeric
Review-Date Time	0	Date and time the review was submitted	Date Time
Rating-Overall	0	Overall rating given by the employee	Numeric
Rating-Work–Life Balance	4,524	Rating given for work–life balance	Numeric
Rating-Culture and Values	4,789	Rating given for company culture and values	Numeric
Rating-Diversity and Inclusion	12,651	Rating given for diversity and inclusion	Numeric
Rating-Senior Leadership	5,164	Rating given for senior leadership	Numeric
Rating-Career Opportunities	4,479	Rating given for career opportunities	Numeric
Rating-Compensation and Benefits	4,455	Rating given for compensation and benefits	Numeric
Current Job	6,362	Indicates whether the employee is still at the company	Boolean
Job Title	0	Title of the employee’s job	Text/String
Summary	74	Employees’ overall evaluation of the company	Text/String
Pros	0	Pros mentioned by the employees in their review	Text/String
Cons	0	Cons mentioned by the employees in their review	Text/String

Data integrity and uniqueness were rigorously verified prior to analysis. A total of 527 duplicate records were excluded to ensure data quality. Notably, the “Diversity and Inclusion” rating contains 12,651 missing values, attributable to the fact that Glassdoor introduced this category only in August 2020 (Wang et al., 2023). Missing numerical ratings across other dimensions were coded as NaN (Not a Number) for subsequent processing.

### 4.2 Employee classification

To address missing job title information, we developed a binary classification model using machine learning algorithms to categorize employees based on review text. Initial labeling distinguished between gig workers (e.g., delivery drivers) and traditional employees based on available job title data. The dataset was partitioned into training (80%) and testing (20%) sets to mitigate model overfitting.

To identify the optimal model, we evaluated two text representation methods (i.e., BoW and TF-IDF) across five standard machine learning algorithms: Logistic Regression (LR), Decision Tree (DT), Random Forest (RF), Support Vector Machine (SVM), and Naive Bayes (NB), yielding 10 distinct classification models. Training results are summarized in Table 2. The TF-IDF representation consistently outperformed BoW. Among the algorithms, the Naive Bayes model achieved superior performance, with accuracy rates of 92.22% (TF-IDF) and 91.08% (BoW).

**Table 2:** Performance comparison of different models for employee classification.

Model	Precision	Recall	F1 Score	Accuracy
TF-IDF + LR	0.92	0.92	0.92	0.92
TF-IDF + DT	0.84	0.84	0.84	0.84
TF-IDF + RF	0.91	0.92	0.91	0.91
TF-IDF + SVM	0.91	0.91	0.91	0.91
TF-IDF + NB	0.92	0.92	0.92	0.92
BoW + LR	0.91	0.91	0.91	0.91
BoW + DT	0.83	0.83	0.83	0.83
BoW + RF	0.92	0.92	0.92	0.92
BoW + SVM	0.88	0.88	0.88	0.88
BoW + NB	0.92	0.92	0.92	0.92

Comparative analysis indicates that our model’s performance surpasses that of similar classification tasks reported in prior literature (e.g., Kobayashi et al., 2018a; Fyffe et al., 2024). Consequently, the TF-IDF + NB combination was selected as the final classifier. The finalized dataset includes 9,971 gig workers and 9,172 traditional employees.

### 4.3 Comparison of job satisfaction

Ratings Independent samples t-tests were conducted to examine differences in job satisfaction between gig and traditional workers. As shown in Table 3, traditional employees reported significantly higher satisfaction across most dimensions, specifically in “Overall Satisfaction,” “Culture and Values,” “Diversity and Inclusion,” “Senior Leadership,” “Career Opportunities,” and “Compensation and Benefits.” Conversely, no statistically significant difference was observed regarding “Work–Life Balance.”

**Table 3:** *T*-test results for the comparison between gig workers and traditional workers in various job satisfaction categories.

Job Satisfaction Dimension	Mean-_Gig Worker_	SD-_Gig Worker_	Mean-_Traditional Worker_	SD-_Traditional Worker_	*t*	*p*
Overall	3.30	1.35	4.04	1.15	40.47	<0.001
Work–Life Balance	3.71	1.48	3.70	1.26	−0.64	0.524
Culture and Values	2.75	1.48	3.92	1.31	49.77	<0.001
Diversity and Inclusion	3.75	1.42	4.15	1.14	12.39	<0.001
Senior Leadership	2.47	1.46	3.60	1.37	47.16	<0.001
Career Opportunities	2.61	1.52	3.84	1.31	52.73	<0.001
Compensation and Benefits	2.51	1.36	3.88	1.19	64.73	<0.001

However, aggregating text data from 2011 to 2025 into a single pool for *t*-tests introduces severe historical bias. To address this, we also conducted year-by-year *t*-tests (omitted here for brevity). The results revealed that a non-significant difference in “Work–Life Balance” between the two groups was only observed in 2014, 2017, 2020, and 2022; across all other years and dimensions, gig workers reported significantly lower satisfaction than traditional employees.

These findings suggest that, with the exception of work–life balance, gig workers experience lower job satisfaction than traditional employees. Furthermore, satisfaction scores among gig workers exhibited greater variability. Both groups reported the highest satisfaction levels in “Diversity and Inclusion” and the lowest in “Senior Leader-ship.”

### 4.4 Analyzing sentiment differences between gig workers and traditional employees

To examine linguistic nuances in employee feedback, we employed the TextBlob library to conduct sentiment analysis on the textual review data. Each review was assigned a polarity score ranging from −1 (extremely negative) to +1 (extremely positive), quantifying the emotional tone of the content. Our analysis revealed statistically significant differences in sentiment scores between gig workers and traditional employees across all three text categories: “Summary,” “Pros,” and “Cons.”

To validate the effectiveness of the sentiment measurement, we randomly sampled 500 employee reviews, which were independently annotated by two coders into three categories (positive, neutral, and negative). TextBlob-based sentiment classification was derived from polarity scores, with polarity > 0.05 classified as positive, polarity < −0.05 as negative, and −0.05 ≤ polarity ≤ 0.05 as neutral. Inter-rater reliability was assessed using Cohen’s Kappa, indicating an extremely high level of agreement between coders (*κ* = 0.958). Moreover, TextBlob classifications showed strong consistency with human judgments (*κ* = 0.866).

Specifically, traditional employees demonstrated significantly higher sentiment scores than gig workers in “Summary” reviews (Δ*M* = 0.16, 95% CI [0.15, 0.17], *t*(18,631.06) = 27.63, *p* < 0.001). This trend persisted in “Pros” reviews (Δ*M* = 0.08, 95% CI [0.08, 0.09], *t*(18,964.13) = 20.51, *p* < 0.001) and “Cons” reviews (Δ*M* = 0.05, 95% CI [0.04, 0.06], *t*(19,034.69) = 12.11, *p* < 0.001). These findings align with our earlier analysis of numerical job satisfaction ratings, indicating that the divergence between gig and traditional workers extends beyond quantitative metrics to qualitative textual expressions. Collectively, the results suggest that gig workers not only assign lower numerical ratings but also articulate more negative sentiments in their online reviews compared to their traditional employee counterparts.

### 4.5 Topic modeling analysis

To develop a comprehensive understanding of the factors contributing to lower job satisfaction and heightened negative sentiments among gig workers, we employed Structural Topic Modeling (STM) to identify latent themes and patterns within the textual data.

#### 4.5.1 Data preprocessing

Text data preprocessing followed the protocol outlined in Section 3.2. We transformed the text into n-grams (i.e., unigrams, bigrams, and trigrams) and converted them into a Bag-of-Words (BoW) representation. To mitigate computational complexity and reduce dimensionality, low-frequency words appearing fewer than five times across the entire corpus were excluded. The final preprocessed corpus comprised 38,064 documents, 3,313 unique terms, and 361,521 tokens.

#### 4.5.2 Determination of optimal topic count

**Figure 1:**
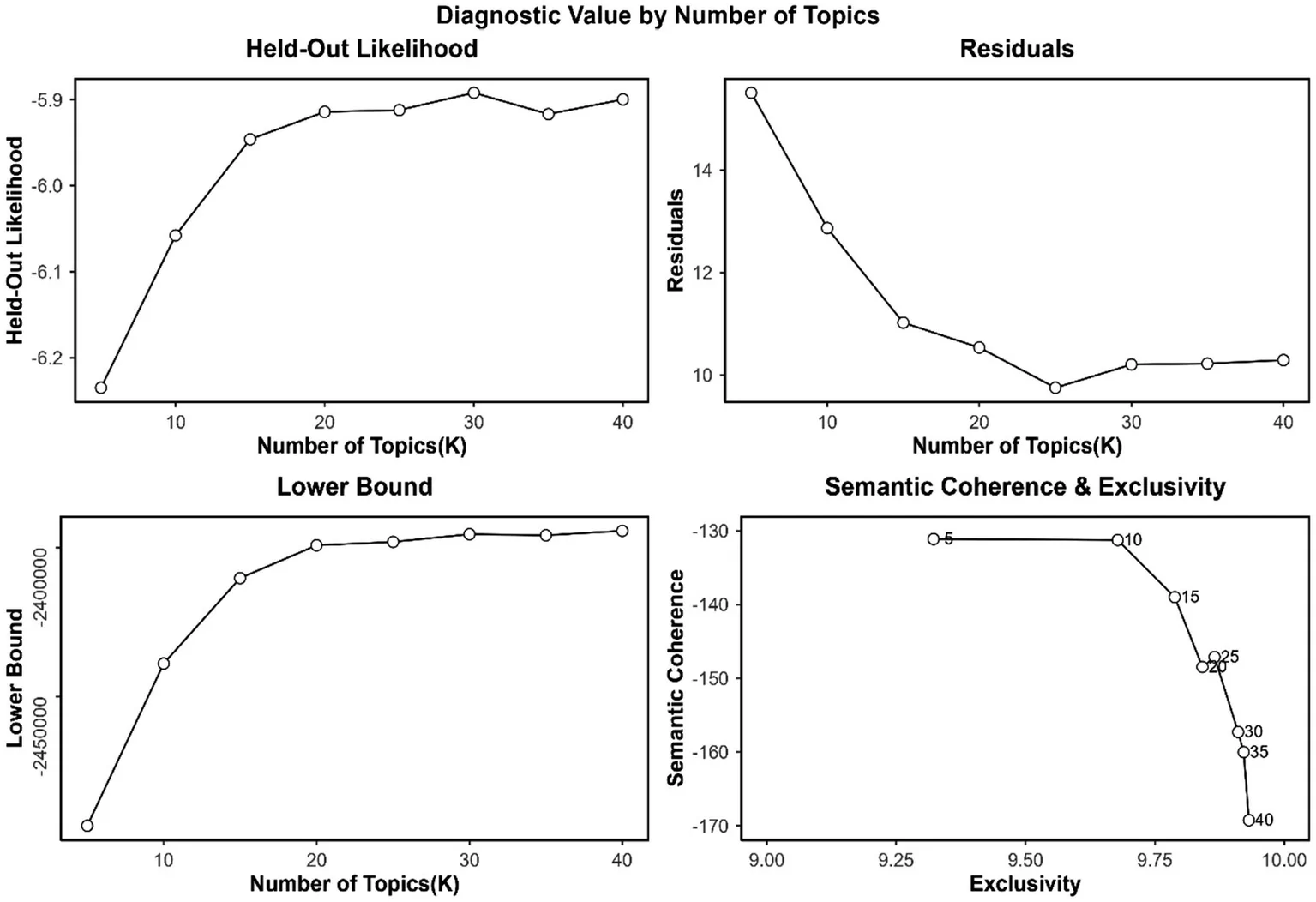
Topic number diagnosis values that span 5–40.

Selecting the optimal number of topics (*K*) is critical for balancing model fit and interpretability. We estimated models with *K* ranging from 5 to 40, evaluating them using held-out likelihood, residuals, lower bounds, semantic coherence, and exclusivity (see Roberts et al., 2019 for diagnostic definitions). As illustrated in Figure 1, fit metrics (held-out likelihood, residuals, and lower bound) improved and stabilized as *K* in-creased, showing substantial gains up to *K* = 25 with marginal improvements thereafter. Concurrently, we examined semantic coherence and exclusivity as indicators of interpretability. While coherence generally decreased as *K* grew, exclusivity tended to increase; at *K* = 25, both measures achieved an optimal balance. Consequently, based on the trade-off between model fit and interpretability, we selected *K* = 25 as the optimal number of topics. This configuration effectively captured the corpus’s underlying structure while maintaining high semantic coherence and exclusivity.

#### 4.5.3 Topic labeling and interpretation

The STM generates two key distributions: (1) a topic–word distribution (probability distribution over words for each topic) and (2) a document–topic distribution (probability distribution over topics for each document). To interpret each topic’s content, we extracted the top five words with the highest probabilities and the top-ranked documents with the highest topic proportions. Based on these lexical items and exemplar reviews, we assigned descriptive labels to each topic. Table 4 reports the top five words, representative documents, and corresponding labels, which are used consistently in subsequent analyses.

**Table 4:** Examples of the highest probability words and documents in each topic.

Topic	Keywords	Explanation	Example
Topic 1: Job Location	Ride, pick, now, area, far	This topic reflects the geographical aspect of work assignments for Uber drivers.	When picking up riders, if the request was 10 + min away, cases might occur wherein you would be 7–8 min into the pickup and Uber would cancel the ride because a closer driver would be found at that time.
Topic 2: Organization Atmosphere	Job, perk, love, awesome, atmosphere	This topic encompasses the appreciation, admiration, and recognition of perks and the work atmosphere.	Salary, perks, atmosphere at work: excellent team
Topic 3: Satisfactory Compensation	Good, pay, part, bonus, side	This topic covers satisfaction with part-time job compensation.	Definitely good money as a part-time job
Topic 4: Corporate Public Image	Company, change, improve, negative, press	This topic includes discussions about the company, changes, improvements, and the company’s public image influenced by media coverage.	The only con for me is the perception of the company in light of all the news articles that may paint Uber in a bad light.
Topic 5: Benefits	Benefit, free, food, lunch, competitive	This topic highlights employee perks, including free food, lunch, and competitive benefits.	Catered lunches daily, Uber credits, Navia benefits, health coverage, maternity and paternity benefits
Topic 6: Workload and Stress	Many, always, quickly, require, stress	This topic represents a fast-paced and demanding work environment, and work-related stress.	Many hours of work sitting behind a steering wheel can be tiring.
Topic 7: Work Pace	Fast, easy, pace, freedom, quick	This topic focuses on a fast-paced work environment with efficient work processes, offering freedom and quickness.	Easy, quick, efficient, and reliable fun
Topic 8: Meeting Different People	People, new, meet, interest, different	This topic relates to social interactions and meeting new individuals with diverse interests.	Work at your own hours, meet great people, explore the city. Meet interesting people. I like adventuring around the city and learning new things.
Topic 9: Controversies of Business Ethics	Business, contractor, lose, cheap, exploitation	This topic reflects controversies that surround the ethical aspects of gig economy business models.	No ethics, whiney entitled trust-fund kid bullying tactics used to get their way in local governments.
Topic 10: Costs and Expenses of Driving	Car, gas, vehicle, wear, tear	This topic highlights the costs and expenses associated with driving, including gas, vehicle maintenance, and wear and tear.	Have to factor in costs of using vehicle (insurance, gas, repairs)
Topic 11: Uncertainties of Work	Get, will, do not, know, never, help, end	This topic represents the unpredictability of work and the presence of uncertainties.	You will probably have a few late nights while getting up to speed.
Topic 12: Challenges Faced by Gig Workers	Driver, customer, low, tip, rate, rider, app, service, delivery, order	This topic highlights the challenges faced by gig workers, including dealing with customers, low wages, tipping rates, app issues, customer service, and delivery orders.	App regularly malfunctioned. Customers’ instructions were frequently vague or flat out wrong. Staff at most apartment buildings were often rude. Customers in most apartment buildings want you to track them down in their workplaces or apartment buildings and do not answer in a timely manner.
Topic 13: Work-Life Balance	Really, bad, life, balance, nothing, poor	This topic reflects employees’ attitudes toward work–life balance.	Low work–life balance (but still better than consulting and investment banking)
Topic 14: Flexible Work Arrangements	Hour, flexible, make, long, flexibility	This topic emphasizes flexible work arrangements.	You can work as long as you want.
Topic 15: No Cons	Just, think, con, none, find	Some employees express the view that they find no negative aspects or disadvantages.	I honestly cannot find a con in my personal experience.
Topic 16: Management and Leadership	Management, change, lack, leadership, structure	This topic is related to the organizational structure, management practices, and leadership approaches within the company.	Lack of communication from management, promotions are based on favoritism.
Topic 17: Flexible Schedule	Work, want, schedule, boss, whenever	This topic reflects the autonomy of work, being able to work whenever desired and being one’s own boss.	Work anytime you want, go offline anytime you want.
Topic 18: Neglect of Employees	Uber, take, care, does not, greedy	The presence of keywords such as “Uber,” “does not,” “take,” and “care” indicates a lack of concern for workers by the company.	Uber Corporation does not care about drivers at all.
Topic 19: Low Wage	Pay, little, wage, tax, minimum	This topic reflects employees’ dissatisfaction with the low level of compensation they receive.	They keep cutting the rate per mile. Pay is now very low, and after expenses, you make less than minimum wage. Gas prices are going up yet they are cutting pay.
Topic 20: Driver Service and Account Issues	Support, issue, call, tell, without, say, account, phone, problem, back	In this topic, employees discuss customer service and account-related issues, specifically those related to their support experience.	Terrible support, no email, no telephone, useless
Topic 21: Additional Income Opportunities	Can, time, income, cash, extra	This topic expresses the opportunity to increase additional income by driving during free time.	Work when you want, can cash out five times daily, and different promotions weekly
Topic 22: Work Environment and Coworkers	Work, environment, nice, everyone, friendly	This topic focuses on the work environment and relationships with coworkers.	It is a very dynamic working place, with many cool people and a great environment.
Topic 23: Culture and Growth Opportunity	Great, culture, opportunity, learn, growth	This topic strongly emphasizes the work culture and opportunities for professional growth.	Amazing work culture, talented professionals, inspiring stakeholders, great platform to showcase and enhance your talents
Topic 24: Working and Rest Time	Day, every, week, night, month	This topic primarily discusses working time and rest time.	I prefer to have weekends off every week.

Our STM analysis yielded coherent and distinctive topics that comprehensively cover all five facets of the Job Descriptive Index (JDI), while also highlighting significant subtopics. Notably, the STM-generated topics incorporate essential factors not explicitly addressed in the JDI (see Table 5). Beyond the traditional five factors of job satisfaction, we identified additional influencers relevant to the gig economy. For instance, topics such as “Costs and Expenses of Driving” and “Driver Service and Account Issues” are particularly salient for gig workers. Furthermore, factors including interpersonal interactions, work–life balance, corporate public perception, business ethics controversies, and work–rest schedules significantly affect employee job satisfaction.

**Table 5:** Comparison of JDI and STM topics.

JDI facets	STM topics
Coworkers	Topic 2: Organization Atmosphere Topic 22: Work Environment and Coworkers
Work Itself	Topic 1: Job Location Topic 6: Workload and Stress Topic 7: Work Pace Topic 11: Uncertainties of Work Topic 12: Challenges Faced by Gig Workers Topic 14: Flexible Work Arrangements Topic 17: Flexible Schedule
Pay	Topic 3: Satisfactory Compensation Topic 5: Benefits Topic 19: Low Wage Topic 21: Additional Income Opportunities Topic 25: Motivation and Incentives
Opportunities for promotions	Topic 23: Culture and Growth Opportunity
Supervision	Topic 16: Management and Leadership Topic 18: Neglect of Employees
Unaddressed Facets in JDI	Topic 4: Corporate Public Image Topic 8: Meeting Different People Topic 9: Controversies of Business Ethics Topic 10: Costs and Expenses of Driving Topic 13: Work–Life Balance; Topic 15: Lack of Cons Topic 20: Driver Service and Account Issues Topic 24: Working Time and Rest Time

#### 4.5.4 Visualization of STM results

Visualization provides an intuitive understanding of the data’s underlying themes and interrelationships. We present the results through topic proportions and metadata relationships.

##### 4.5.4.1 Topic prevalence

**Figure 2:**
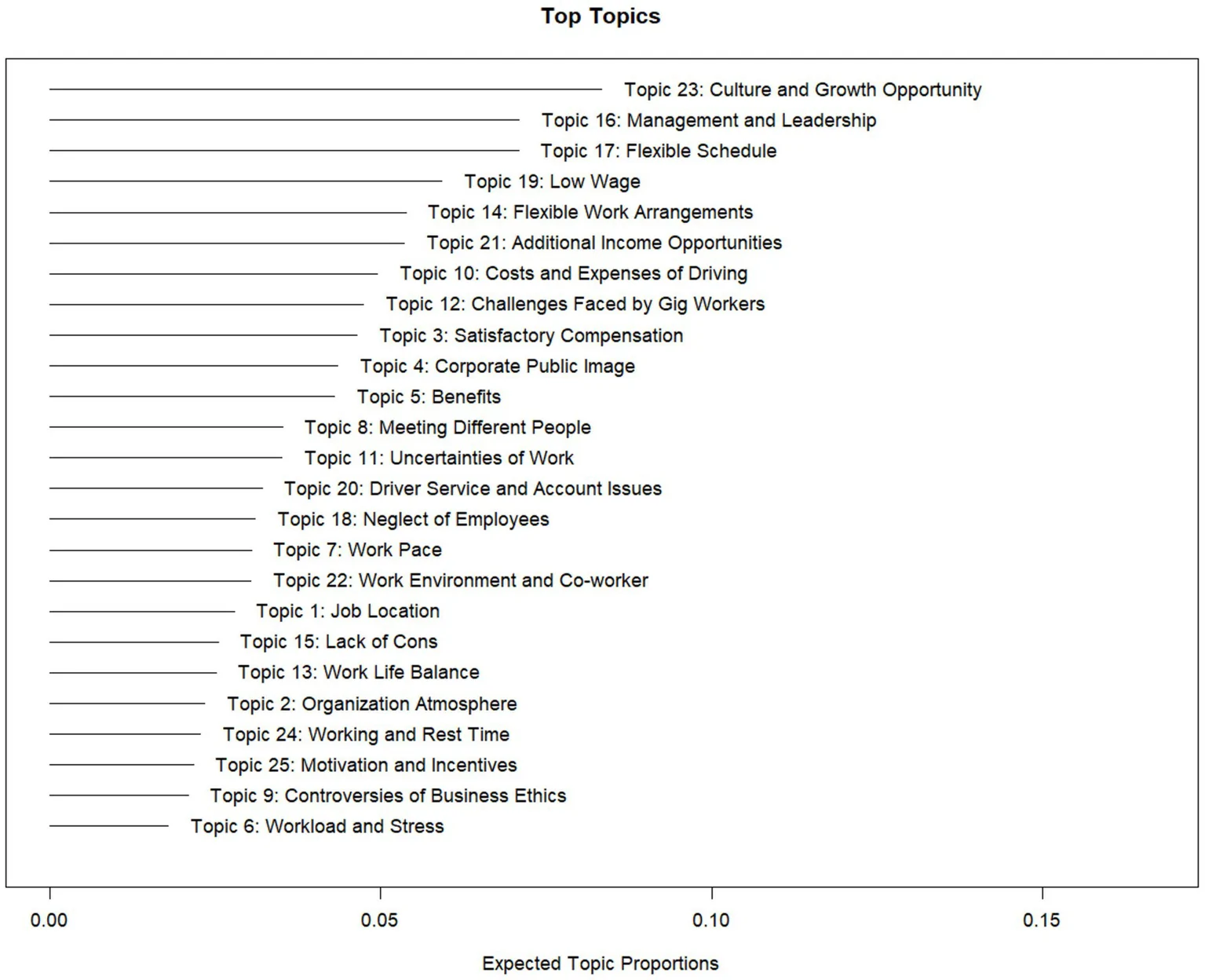
Expected proportions of the 25 topics.

We first examined the overall prevalence of the 25 topics within employee comments. As shown in Figure 2, Topic 23 (“Culture and Growth Opportunity”) exhibited the highest expected proportion, followed by Topic 16 (“Management and Leadership”) and Topic 17 (“Flexible Schedule”). In contrast, Topic 6 (“Workload and Stress”), Topic 9 (“Controversies of Business Ethics”), and Topic 25 (“Motivation and Incentives”) appeared relatively infrequently in the corpus.

##### 4.5.4.2 Topic relationships with metadata

Beyond topic prevalence, we investigated employee attitudes toward these topics and explored differences in topic focus between gig and traditional workers. Specifically, we analyzed which topics were more commonly discussed by each group, which received similar attention, and how these topics varied over time and with satisfaction levels. Given that employees frequently discuss multiple topics within a single review, identifying co-occurring topics is essential. STM offers a framework for examining the influence of covariates on topic prevalence through a generalized linear model. To analyze these relationships, we included three covariates: review type (Pros vs. Cons) and job type (Gig Worker vs. Traditional Worker). Equation 1 represents the relationship between topic prevalence and the covariates:

Prevalence=g(jobtype,review type) (1)

**Figure 3:**
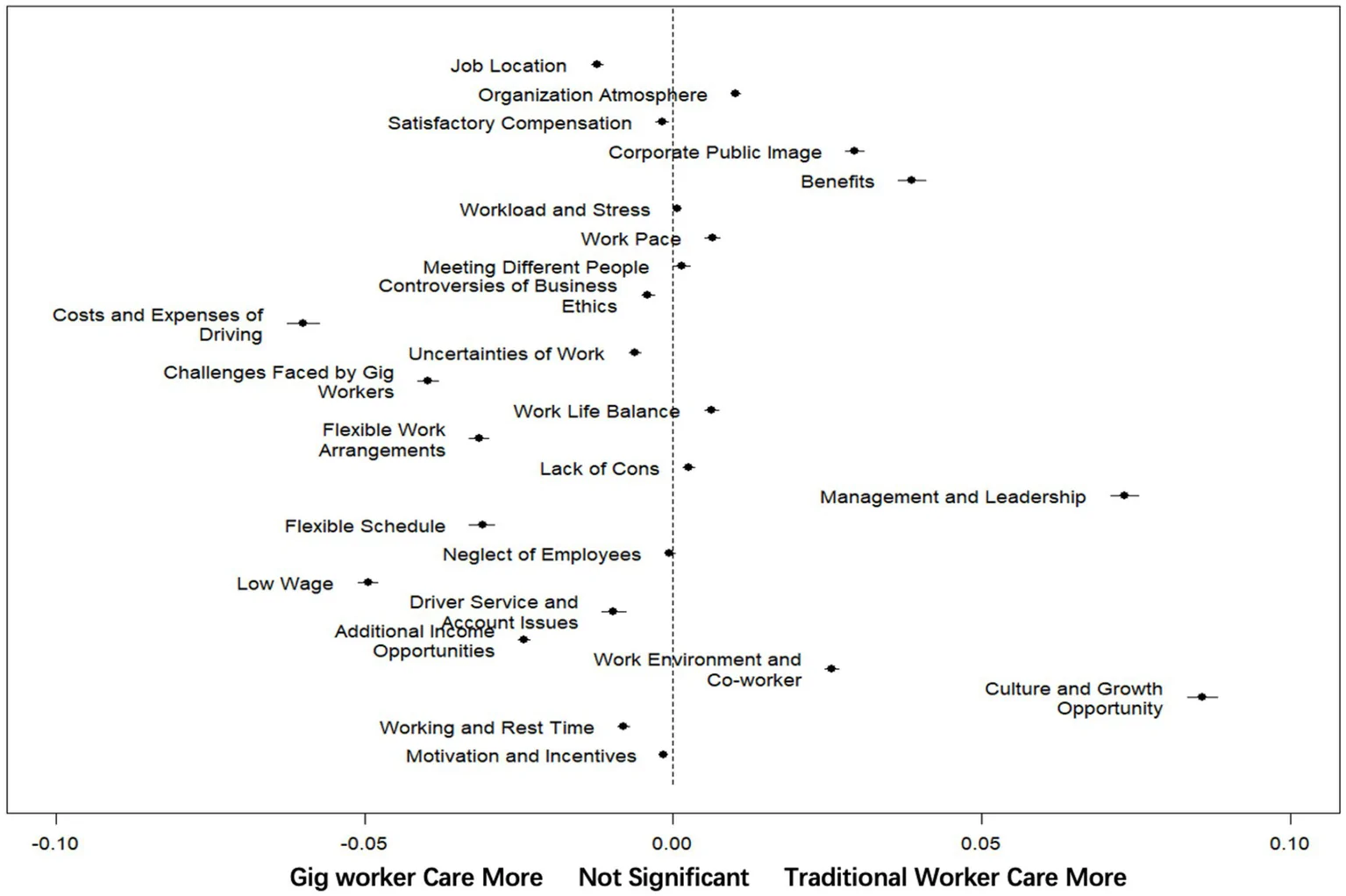
Topical prevalence contrast between gig workers and traditional workers. The “dots” in the figure depict the difference in topic prevalence between the two groups estimated by a point estimate, while the “lines” on the point represent the corresponding 99% CI of that difference. Specifically, if CI encompasses the value of 0.00, then the difference is not deemed to be statistically significant.

Focusing on job type (Figure 3), we found that gig and traditional workers shared similar attention levels only regarding “Workload and Stress,” “Meeting Different People,” and “Neglect of Employees.” The remaining 22 topics differed significantly. Traditional workers placed greater emphasis on “Culture and Growth Opportunity,” “Management and Leadership,” “Benefits,” “Work Environment and Coworkers,” “Corporate Public Image,” “Organizational Atmosphere,” “Work–Life Balance,” and “Work Pace,” and were more likely to report a “Lack of Cons,” consistent with their higher overall satisfaction. Conversely, gig workers focused more on the remaining 13 topics, including “Costs and Expenses of Driving,” “Challenges Faced by Gig Workers,” and “Low Wage.”

**Figure 4:**
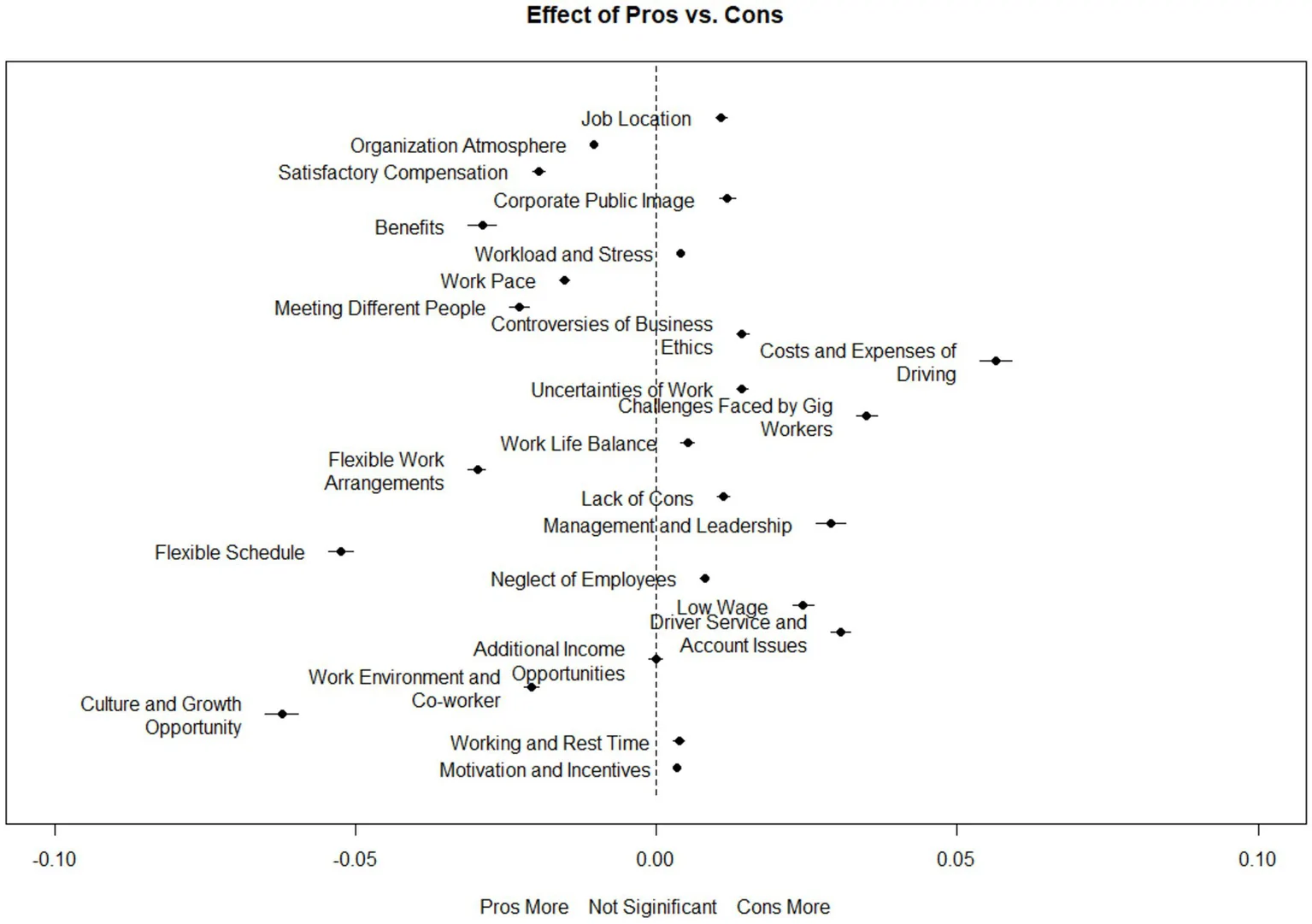
Topical prevalence contrast between pros and cons.

We further compared topic prevalence between Pros and Cons reviews. Figure 4 displays the contrast, where “Additional Income Opportunities” exhibited no significant difference. Compared to Cons reviews, Pros reviews were more inclined to report positive aspects such as “Organizational Atmosphere,” “Satisfactory Compensation,” “Benefits,” “Work Pace,” “Meeting Different People,” “Flexible Work Arrangements,” “Flexible Schedule,” “Work Environment and Coworkers,” and “Culture and Growth Opportunity,” indicating higher satisfaction with these dimensions. In Cons reviews, employees tended to express dissatisfaction regarding “Job Location,” “Corporate Public Image,” “Workload and Stress,” “Work Pace,” “Controversies of Business Ethics,” “Costs and Expenses of Driving,” “Uncertainties of Work,” “Challenges Faced by Gig Workers,” “Work–Life Balance,” “Lack of Cons,” “Management and Leadership,” “Neglect of Employees,” “Low Wage,” “Driver Service and Account Issues,” “Work Time and Rest Time,” and “Motivation and Incentives.”

## 5 Discussion

This study sought to examine the disparities in job satisfaction between gig workers and traditional employees by leveraging a large-scale dataset of Glassdoor reviews and ratings. Employing advanced machine learning techniques—including classification modeling, sentiment analysis, and Structural Topic Modeling (STM)—we uncovered nuanced differences in how these two groups experience and articulate their work lives. The findings indicate that gig workers generally report lower job satisfaction and articulate more negative sentiments compared to their traditional employee counter-parts. Furthermore, the structural dimensions influencing job satisfaction diverge significantly between the two groups.

Our STM analysis identified 25 distinct factors influencing employee job satisfaction, encompassing all five facets of the Job Descriptive Index (JDI) alongside additional significant subtopics specific to the gig economy. We observed notable variations in the prevalence of these topics. “Culture and Growth Opportunity” emerged as the most frequently discussed theme, followed by “Management and Leadership” and “Flexible Schedule.” In contrast, topics such as “Workload and Stress,” “Controversies of Business Ethics,” and “Motivation and Incentives” received comparatively less attention. This analysis provides critical insights into the primary concerns articulated in employee feedback and highlights the relative significance of different topics in shaping overall job satisfaction.

To further dissect the differences in job satisfaction factors, we integrated metadata into the STM analysis. The results revealed distinct patterns in topic attention. Gig workers demonstrated a stronger focus on 13 topics, including “Costs and Expenses of Driving,” “Challenges Faced by Gig Workers,” and “Low Wage.” Conversely, traditional employees placed greater emphasis on nine topics, such as “Culture and Growth Opportunity,” “Management and Leadership,” and “Work Environment and Coworkers.” Notably, no significant difference in attention was observed between the two groups for “Workload and Stress,” “Meeting Different People,” and “Neglect of Employees.” Furthermore, our analysis indicated that gig workers rarely expressed experiences related to traditional JDI dimensions such as “Coworkers,” “Opportunities for Promotion,” and “Supervision.” This suggests that the determinants of job satisfaction for gig workers operate under a different structural logic than those for traditional employees.

Additionally, we analyzed the distribution of these topics across “Pros” and “Cons” reviews to deepen our understanding of the drivers of satisfaction and dissatisfaction. Topics associated with gig workers were predominantly prevalent in the “Cons” category (10 cons topics, 1 neutral, 2 pros), whereas topics associated with traditional employees were more common in the “Pros” category (8 pros topics, 4 cons). These findings underscore the distinct perspectives and emotional expressions conveyed by each group: gig workers tend to express more concerns and dissatisfaction, while traditional employees emphasize positive aspects of their work experiences. These observations align with our statistical analysis of job satisfaction ratings and sentiment analysis results. Collectively, the findings suggest that platform-based employment not only depresses average job satisfaction but also fundamentally reshapes its underlying structural dimensions.

### 5.1 Theoretical implications

First, this study demonstrates the potential and advantages of utilizing big data from online review platforms to investigate employee job satisfaction. By harnessing vast amounts of user-generated textual data, this research expands the application of big data analytics within the field of management. The analysis of online reviews offers a unique opportunity to gain deep insights into employee experiences and sentiments, transcending the limitations of traditional survey methods, such as recall bias and social desirability effects. This advancement contributes to the broader field of management research by offering new avenues for understanding organizational phenomena. Employing big data analysis facilitates a comprehensive understanding of the factors influencing job satisfaction. Through the examination of online reviews, the current study not only substantiates the established dimensions of the JDI but also extends them, offering a broader perspective on the determinants of job satisfaction in the contemporary workplace.

Second, this study highlights the value of integrating multiple analytical approaches—including statistical analysis and machine learning techniques (classification modeling, sentiment analysis, and topic modeling)—to deepen the theoretical understanding of employee job satisfaction. This integrated strategy enables the simultaneous examination of different dimensions of job satisfaction and uncovers underlying patterns in large-scale review data, offering a comprehensive view across diverse work settings. The convergence of findings across methods strengthens the robustness of our conclusions. For instance, statistical analysis showed that gig workers reported higher satisfaction with “Work–Life Balance,” consistent with topic modeling results where “Flexible Work Arrangements” and “Flexible Schedule” emerged as salient positive themes. Similarly, traditional employees reported lower satisfaction with “Senior Leadership,” aligning with cons-related reviews clustering around “Management and Leadership.” Sentiment analysis revealed more negative affect among gig workers, mirroring their higher concentration of cons topics in the STM results. This cross-method consistency not only validates the reliability and applicability of machine learning techniques in management research but also illustrates how advanced analytics can be used to explore complex organizational phenomena and extract theoretically meaningful insights from unstructured text. By leveraging these techniques in a big data context, our study expands the methodological toolkit for job satisfaction research and responds to calls for more innovative, data-intensive approaches in management studies (George et al., 2016).

Third, the gig economy has reshaped the traditional employment landscape, necessitating a deep understanding of gig workers’ job satisfaction. The current study addresses a significant research gap by investigating differences in job satisfaction between gig workers and traditional employees, contributing to the theoretical understanding of job satisfaction in diverse employment contexts. Through an analysis of Glassdoor reviews and ratings, we gain valuable insights into the factors influencing job satisfaction in these distinct employment arrangements. In doing so, the current study expands the existing literature by uncovering the unique experiences, challenges, and drivers of satisfaction specific to gig workers and traditional employees. These findings offer valuable contributions to our theoretical understanding of job satisfaction dynamics in the evolving world of work, ultimately assisting organizations, policymakers, and researchers in comprehending and addressing the job satisfaction needs of gig workers.

### 5.2 Practical implications

First, the insights gained from this study can inform human resource practices and policies aimed at enhancing job satisfaction. Organizations can use the identified factors to tailor their strategies and initiatives to meet the specific needs and preferences of gig workers and traditional employees. By understanding the distinct perspectives of the two groups, organizations can develop targeted interventions and practices to improve job satisfaction and, ultimately, enhance overall organizational performance. For platform companies, this might involve redesigning incentive structures to address the specific “Costs and Expenses” concerns of gig workers, while traditional firms might focus on enhancing “Culture and Growth Opportunities” for traditional staff.

Second, the findings of this study can benefit individual workers, both gig and traditional, by providing an improved understanding of the factors that contribute to job satisfaction. This knowledge can empower workers to make informed decisions about their career choices and work arrangements, considering the potential effects on their job satisfaction. It can also help workers identify areas for improvement or negotiation in their current work situations to enhance their well-being. For policymakers, these findings highlight the need for regulatory frameworks that address the unique vulnerabilities of gig workers, such as income instability and lack of benefits, to ensure a more equitable labor market.

### 5.3 Limitations and future research

This study entails several notable limitations, which also point to promising directions for future scholarly inquiry. First, the primary limitation concerns the scope and cross-context generalizability of the empirical data. While this study adopts a large longitudinal dataset consisting of 19,610 consumer reviews, the textual corpus of gig workers is exclusively collected from the Uber platform. Accordingly, the identified antecedents of worker dissatisfaction, including vehicle-related operational costs and platform dispatch algorithms, are uniquely indicative of location-dependent, asset-intensive gig economy business models. Hence, the research findings cannot be directly generalized to web-based, asset-light gig work modalities, represented by digital micro-tasking and knowledge-based freelance services. To develop a comprehensive classification system for gig work satisfaction, subsequent studies could broaden the empirical scope via cross-platform comparative analysis across heterogeneous platform types, for instance, contrasting ride-hailing drivers with freelance graphic designers registered on the Upwork marketplace.

Second, this research is constrained by the exclusion of work volition as a key boundary condition. Defined as the extent to which workers autonomously choose to engage in a given work arrangement, work volition constitutes a core occupational psychological construct (Duffy et al., 2012). Cumulative evidence from organizational psychology literature has validated work volition as a pivotal predictor of workplace attitudes. Specifically, gig workers who participate in platform work out of personal preference for work flexibility manifest higher job satisfaction and weaker turnover intentions, relative to those involuntarily engaging in gig work due to structural labor market barriers or financial hardship. Given that the analytical framework draws on large-scale anonymous user-generated reviews retrieved from Glassdoor, this study fails to quantitatively assess individual workers’ baseline work volition and pre-gig employment status via psychometric measurement. As such, the documented lower overall satisfaction among gig workers may be skewed by the subgroup of involuntary gig workers trapped in structural underemployment. Future research adopting questionnaire surveys or mixed-method methodologies should incorporate work volition as a moderating variable to unpack heterogeneous psychological contracts among gig economy participants.

Third, the original dataset lacks granular demographic information of review contributors. Individual attributes encompassing age, educational attainment and other personal traits have been well documented to shape job satisfaction levels. Follow-up research may advance this field by integrating review data with external demographic databases, to unpack the demographic heterogeneity in gig workers’ job satisfaction patterns.

Fourth, prevalent machine learning models suffer from inherent black-box limitations. In this study, the machine learning model deployed for worker attitude categorization lacks interpretability regarding its internal classification mechanisms. Future research is recommended to adopt interpretable machine learning tools (e.g., LIME, SHAP) to demystify model decision-making logics and improve analytical fairness in gig work textual analysis.

Finally, future studies may integrate advanced natural language processing (NLP) techniques, including Word2Vec, BERT and other state-of-the-art word embedding algorithms, with conventional topic modeling approaches. Such integrated analytical frameworks can strengthen model semantic comprehension, and yield finer-grained interpretations of contextual implications embedded in worker textual feedback.

## Data Availability

Publicly available datasets were analyzed in this study. The empirical data supporting the findings of this study were retrieved from public company reviews on Glassdoor (https://www.glassdoor.com/reviews/uber-reviews-e575263.htm). The dataset includes employee reviews and ratings. To comply with legal and ethical requirements regarding the use of web-scraped data, the complete raw dataset has not been deposited in a public repository. However, the data collection and analysis followed the methodological framework and NLP processing scripts demonstrated by Guo et al. (2024).

## Footnotes

1
https://www.glassdoor.com/reviews/uber-reviews-e575263.htm
